# Inhibition of Intestinal Epithelial Apoptosis Improves Survival in a Murine Model of Radiation Combined Injury

**DOI:** 10.1371/journal.pone.0077203

**Published:** 2013-10-28

**Authors:** Enjae Jung, Erin E. Perrone, Pavan Brahmamdan, Jacquelyn S. McDonough, Ann M. Leathersich, Jessica A. Dominguez, Andrew T. Clark, Amy C. Fox, W. Michael Dunne, Richard S. Hotchkiss, Craig M. Coopersmith

**Affiliations:** 1 Department of Surgery, Washington University School of Medicine, St. Louis, Missouri, United States of America; 2 Department of Anesthesiology, Washington University School of Medicine, St. Louis, Missouri, United States of America; 3 Department of Pathology and Immunology, Washington University School of Medicine, St. Louis, Missouri, United States of America; 4 Department of Basic Sciences, Bastyr University California, San Diego, California, United States of America; 5 The Emory Center for Critical Care and Department of Surgery, Emory University School of Medicine, Atlanta, Georgia, United States of America; University of Cincinnati, United States of America

## Abstract

World conditions place large populations at risk from ionizing radiation (IR) from detonation of dirty bombs or nuclear devices. In a subgroup of patients, ionizing radiation exposure would be followed by a secondary infection. The effects of radiation combined injury are potentially more lethal than either insult in isolation. The purpose of this study was to determine mechanisms of mortality and possible therapeutic targets in radiation combined injury. Mice were exposed to IR with 2.5 Gray (Gy) followed four days later by intratracheal methicillin-resistant *Staphylococcus aureus* (MRSA). While either IR or MRSA alone yielded 100% survival, animals with radiation combined injury had 53% survival (p = 0.01). Compared to IR or MRSA alone, mice with radiation combined injury had increased gut apoptosis, local and systemic bacterial burden, decreased splenic CD4 T cells, CD8 T cells, B cells, NK cells, and dendritic cells, and increased BAL and systemic IL-6 and G-CSF. In contrast, radiation combined injury did not alter lymphocyte apoptosis, pulmonary injury, or intestinal proliferation compared to IR or MRSA alone. In light of the synergistic increase in gut apoptosis following radiation combined injury, transgenic mice that overexpress Bcl-2 in their intestine and wild type mice were subjected to IR followed by MRSA. Bcl-2 mice had decreased gut apoptosis and improved survival compared to WT mice (92% vs. 42%; p<0.01). These data demonstrate that radiation combined injury results in significantly higher mortality than could be predicted based upon either IR or MRSA infection alone, and that preventing gut apoptosis may be a potential therapeutic target.

## Introduction

The possibility of large populations being exposed to whole-body ionizing radiation (IR) is unfortunately a real concern in our current world condition. IR may result from dispersal of radioactive materials by explosives (i.e. “dirty bomb”), detonation of nuclear devices, or even attacks on commercial nuclear facilities. In addition, with increasing use of nuclear energy facilities, there is also a possibility of accidental exposure following a facility malfunction or natural disaster. The impact of an intentional or accidental nuclear event could be catastrophic – over 100,000 people died in Hiroshima and Nagasaki [1] and it is estimated that detonation of a 20-megaton warhead would kill 2 million people [2]. Current U.S. government planning is centered around a 10 kiloton blast [3], which although smaller, would still assuredly be devastating.

When facing possible terrorist threat or industrial accidents, people may not only face radiation exposure, but they may also have associated trauma, burn, and blast injuries [4]. These concurrent injuries, defined as “radiation combined injuries” often worsen morbidity and mortality in animal models [5]–[9]. In addition to concurrent injuries, patients exposed to IR would also be at increased risk for developing infections due to increased host susceptibility to systemic infections from both endogenous and exogenous organisms [10], [11]. The mechanisms of mortality resulting from radiation combined injury are just beginning to be understood, and relatively few potential therapeutic targets exist [7]. This is of potential great significance since it has been estimated that based upon the atomic bombings of Hiroshima and Nagasaki, radiation combined injury would cause up to 65% of all injuries observed in a future air burst nuclear detonation [8].

The few studies performed on radiation combined injury involving infection as the second insult have used common gram negative organisms such as *Klebsiella pneumoniae* or enteric contents using cecal ligation and puncture [4], [5], [12], [13] However, a recent National Institutes of Health consensus meeting explicitly stated that staphylococcal infections should be anticipated in the wake of a radiation event and recommended further study of IR followed by this common gram positive organism [14]. While staphylococcal infections can be sensitive or resistant to methicillin, the incidence of methicillin-resistant *Staphylococcus aureus* (MRSA) is becoming increasingly common [15]. Nearly 100,000 people are diagnosed with invasive MRSA annually, associated with increased costs and mortality [16], [17], and hospitalized patients from recent radiation accidents have acquired MRSA [18].

Although there is vast experience in treating infections in neutropenic patients, the pathophysiology of radiation combined infection is unique and cannot be inferred from experiences with patients who are neutropenic from other causes [19]. This study examined potential mechanisms of mortality and therapeutic targets in a novel murine model of radiation combined injury using MRSA.

## Materials and Methods

### Animals

All studies were performed using 6 to 12 week-old FVB/N mice or age matched *Fabpl*-Bcl-2 mice that overexpress the anti-apoptotic protein Bcl-2 in their intestinal epithelium on the same genetic background [20]. Mice were kept on a 12 hour light-dark cycle and received non-acidified water and mouse chow *ad libitum*. All experiments were performed in accordance with the National Institutes of Health Guidelines for the Use of Laboratory Animals and were approved by the Institutional Animal Care and Use Committee at Washington University School of Medicine (Protocol 20030265, no longer active since senior author moved institutions) and Emory University School of Medicine (Protocol DAR-200591-032814BN). All surgery (described below) was performed under isoflurane anesthesia and all efforts were made to minimize animal suffering by treating all animals with buprenex post-operatively. For non-survival studies, animals were sacrificed 24 hours following MRSA or sham operation (detailed below) unless a different timepoint is explicitly noted in the results. Animals sacrificed at predetermined endpoints for non-survival studies were sacrificed using asphyxiation by CO2 or via exsanguination under deep isoflurane anesthesia. A different subset of animals were followed for 7 days after MRSA or sham operation for survival studies. Animals were checked twice daily. Moribund animals were sacrificed using humane endpoints. The below criteria were used to identify moribund animals: a) major organ failure or medical conditions unresponsive to treatment such as severe respiratory distress, icterus, uremeia, intractable diarrhea, or self-mutilation, b) surgical complications unresponsive to immediate intervention (bleeding, infection, wound dehiscence) or c) clinical or behavioral signs unresponsive to appropriate intervention persisting for 24 hours including significant inactivity, labored breathing, sunken eyes, hunched posture, piloerection/matted fur, one or more unresolving skin ulcers, and abnormal vocalization when handled. Non-moribund animals that died during the course of the survival curve died as a direct result of their septic insult. Animals that survived 7 days after MRSA or sham operation were sacrificed at the conclusion of this experiment using asphyxiation by CO2.

### Radiation Combined Injury Model

Mice were randomized to receive either irradiation (IR) or no radiation (NR) followed by either MRSA pneumonia or sham operation (sham) four days later. Mice randomized to IR were given whole-body ^137^Cesium-γ radiation at 2.5 Gy at a dose rate of 100 cGy/min). NR mice were treated identically to mice given IR (i.e. placed in the radiator for a similar length of time as animals) except no IR was administered.

MRSA strain 313 was isolated from a patient in the BJC HealthCare system (St. Louis, MO) [21], [22]. This strain has multilocus sequence type 5, staphylococcal cassette chromosome II, and is negative for Panton-Valentine leukocidin. To prepare the inocula, MRSA was grown overnight at 37°C in trypticase soy broth (Becton Dickinson, Sparks, MD), centrifuged at 6,000 g, washed in sterile saline twice and resuspended in sterile saline to a final density of 0.5 at 600 nm, which corresponds to approximately 5×10^8^ CFU/ml.

MRSA pneumonia was induced under isoflurane anesthesia. A midline cervical incision was made and MRSA was injected intratracheally via a 29-gauge insulin syringe. Animals were given an inocula containing 20 µl (1×10^7^ CFU) MRSA. To enhance aspiration of the bacteria, mice were held vertically for 10 seconds [23], [24]. Sham operated mice were treated identically except they received saline instead of MRSA. The midline incision was closed with a 5-0 nylon suture. All mice received 1 ml of saline subcutaneously following the operation.

### Cultures

Bacterial colony counts were measured from both blood and bronchoalveolar lavage (BAL) samples from the same animals. BAL fluid was obtained by lavaging the lungs with 1 ml of sterile saline. Samples were serially diluted in sterile saline and grown on blood agar plates at 37°C. Colonies were counted after 48 hours of incubation. Growth was calculated as CFU/ml, and log transformation of these measurements was used for statistical analysis [25].

### Cytokine Analysis

Whole blood collected at the time of sacrifice was centrifuged to obtain plasma. Plasma and BAL samples were analyzed in duplicates using the Bio-plex Cytokine Pro assay (Biorad, Hercules, CA) per manufacturer’s recommendations to measure concentrations of IL-1β, IL-6, IL-10, IL-13, TNFα, and G-CSF.

### Complete Blood Count Analysis

Blood samples were collected on the same animal both 4 days after IR (just prior to MRSA inoculation) and 24 hours after MRSA pneumonia. Complete blood counts with differentials were performed on a HemaVet 950 hematology analyzer (Drew Scientific, Dallas, TX).

### Intestinal Epithelial Cell Apoptosis

Intestinal epithelial cell apoptosis was identified and quantified using both morphologic and functional approaches by assaying both H&E and caspase-3 staining in the crypts of 100 well-oriented crypt-villus units [26]–[28]. H&E-stained jejunal sections were evaluated for characteristic morphological changes of apoptosis including cell shrinkage with condensed nuclei and nuclear fragmentation. For active caspase-3 staining [29], paraffin-embedded sections were deparaffinized, rehydrated, and then blocked of endogenous peroxidase activity with 30% H_2_O_2_ in methanol for 10 minute. Sections were heated for 45 minutes while immersed in antigen decloaker solution (Biocare Medical), blocked for 30 minutes with 20% goat serum (Vector Laboratories; Burlingame, CA) and then incubated with rabbit polyclonal anti-active caspase-3 antibody (1∶100 diluted in PBS; Cell Signaling Technology; Danvers, MA) overnight at 4°C. The following day, sections were incubated at room temperature with goat anti-rabbit biotinylated secondary antibody (1∶200 diluted in PBS; Vector Laboratories) for 30 minutes. Sections were then incubated for 30 minutes with Vectastain Elite avidin-biotin-peroxidase complex reagent (Vector Laboratories), developed with diaminobenzidine, and counterstained with hematoxylin.

### mRNA Levels of Intestinal Apoptotic Mediators

Total RNA was isolated from jejunal tissue using the RNeasy Mini Kit (Qiagen, Santa Clarita, CA) according to the manufacturer’s protocol. The integrity of RNA was verified by electrophoresis on a 1.2% agarose gel that contained 2.2 M formaldehyde in 1×3-(N-morpholino)propanesulfonic acid buffer (3-(N-morpholino)propanesulfonic acid, pH 7.0; 10 mM sodium acetate; 1 mM EDTA, pH 8.0). Gene expression was evaluated via real-time polymerase chain reaction (PCR) [30]. cDNA was synthesized from 0.5 µg of total RNA. Bid, Bax, Bcl-2, Bcl-x_L_, FADD, FAS, and TRADD mRNA levels were detected using predeveloped TaqMan primers and probes (Applied Biosystems, Foster, CA) and run on the ABI 7900HT Sequence Detection System (Applied Biosystems). Samples were run in duplicate and normalized to expression of the endogenous control, glyceraldehydes-3-phosphate (Applied Biosystems). Relative quantification of PCR products were based upon the value differences between the target gene and glyceraldehydes-3-phosphate using the comparative C_T_ method.

### Villus Length

Villus length was measured in twelve well-oriented jejunal crypt-villus units in micrometers from the crypt neck to the villus tip on H&E-stained sections using MetaMorph Version 7.1.2.0 (Downingtown, PA).

### Intestinal Proliferation

Intestinal epithelial cell proliferation was assessed by quantifying S-phase cells in the crypts of 100 well-oriented jejunal crypt-villus units. In order to label S-phase cells, mice were injected 90 minutes prior to sacrifice with 5-bromo-2′deoxyuridine (BrdU) (5 mg/mL diluted in saline; Sigma; St. Louis, MO) [31]. BrdU was detected in jejunal sections via immunohistochemistry using a commercially available kit (BD PharMingen, San Diego, CA).

### Absolute Lymphocyte Cell Counts

Splenocytes were dissociated by gently pressing through a 70 micron filter and then washed with FACS buffer. The total number of viable cells (based upon exclusion of trypan blue) was determined using a Beckman-Coulter Cell Counter. Splenocytes were also stained with fluorochrome-conjugated antibodies to cell subset-specific surface markers CD4, CD8, B220, CD11c, DX5, F480, CD3, and CD20 and the percentage of the different lymphocyte subset was determined by flow cytometric analysis (BD FACScan).

### Lymphocyte Apoptosis

Lymphocyte apoptosis was identified on splenocytes via flow cytometry for active caspase-3 and TUNEL staining. Cells were fixed in 1% paraformaldehyde for 30 minutes at room temperature, washed with FACS buffer, and permeabilized with 90% methanol on ice for 30 minutes. Active caspase-3 was quantitated per manufacturer’s recommendations using primary antibodies to the cleaved fragment of caspase-3 (Cell Signaling) and secondary PE-labeled donkey anti-rabbit IgG antibody to detect the primary antibody. The TUNEL method was performed using a commercially available Apo-BrdU Kit (Phoenix Flow Systems, Inc.; San Diego, CA) per manufacturer instructions. A secondary anti-biotin PE-labeled antibody was used for detecting BrdU-labeled strand breaks.

### Lung Histology and Edema

H&E-stained lung sections were used to qualitatively evaluate the severity of pneumonia using a previously described subjective grading scale by a pathologist blinded to sample identity [23]. Pneumonia severity was graded in each specimen on a scale ranging from 0 to 4 (no histopathologic abnormality to most severe pneumonia).

Pulmonary edema was quantified using wet to dry lung ratio. Wet weight was obtained immediately after sacrifice when both lungs were weighed after removal from the body. Dry weight was obtained after the lungs were dried for 48 hours at 80°C at which time a wet to dry weight ratio was calculated.

### Lung Myeloperoxidase (MPO) Activity

Myeloperoxidase (MPO) activity was assayed as a measurement of neutrophil infiltration. Following sacrifice, lungs were harvested and snap frozen until the time of the assay. Lungs were homogenized in 50 mM sodium phosphate buffer containing 0.5% hexadecyltrimethylammonium bromide, heated for 2 hours at 55°C, then centrifuged at 13,000 rpm for 20 minutes at 4°C. Supernantants were used to measure MPO activity spectrophotometrically at 460-nm wavelength over 6 minutes with readings every 30 seconds (Bio-Tek Instruments-µQuant Microplate Spectrophotometer) using substrate buffer containing o-dianisidine and 0.03% hydrogen peroxide. MPO activity was expressed as change in optical density (OD) per minute per milligram of lung tissue.

### Statistical Analysis

Kruskal-Wallis ANOVA with Dunn’s Multiple Comparison Post-Test was used when 3 or more groups were compared. After determining the groups did not have Gaussian distributions, the Mann-Whitney test was used when only 2 groups were compared. The logrank test was used to analyze survival curves. All data were analyzed using the statistical software program Prism 4.0 (GraphPad Software, San Diego, CA) and are presented as mean ± SEM. P values<0.05 were considered to be statistically significant.

## Results

In order to identify possible synergistic effects of radiation combined injury and distinguish these from the independent effects of either IR or MRSA pneumonia alone, initial experiments were performed in four experimental groups: a) IR followed by sham operation (IR/Sham), b) no radiation with MRSA pneumonia in isolation (NR/MRSA), c) IR followed by MRSA (IR/MRSA) and d) no radiation with sham operation in isolation (NR/Sham). In each group, MRSA pneumonia or sham pneumonia was induced 4 days after IR (or NR).

### Radiation Combined Injury Increases Mortality Compared to IR or MRSA Pneumonia in Isolation

**Figure 1:**
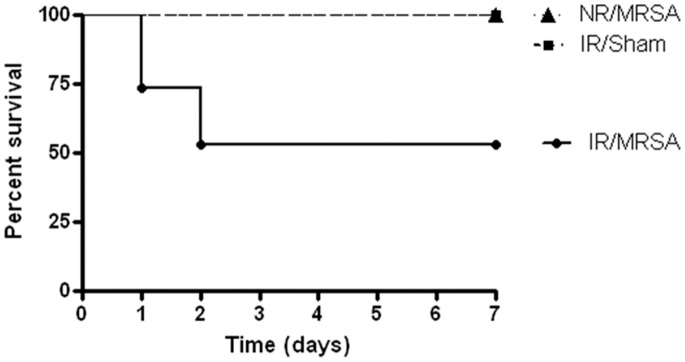
Radiation combined injury increases mortality compared to IR or MRSA alone. Mice that received either 2.5% survival. In contrast, when IR was followed four days later by MRSA, survival was only 53% (n = 8−15/group; p = 0.01). Note that day 0 in this survival curve represents when MRSA or sham pneumonia was induced, and IR was given four days earlier.

Three cohorts of mice were followed 7 days after MRSA pneumonia or sham pneumonia: a) IR/sham, b) NR/MRSA and c) IR/MRSA. Both single injury groups (IR/sham and NR/MRSA) had 100% survival. However, radiation combined injury resulted in only 53% survival (p = 0.01, Fig. 1). Of note, NR/sham mice were not followed for survival since it was expected that sham operation would result in 100% survival.

### Radiation Combined Injury Increases Local and Systemic Bacterial Burden Compared to MRSA Pneumonia Alone

**Figure 2:**
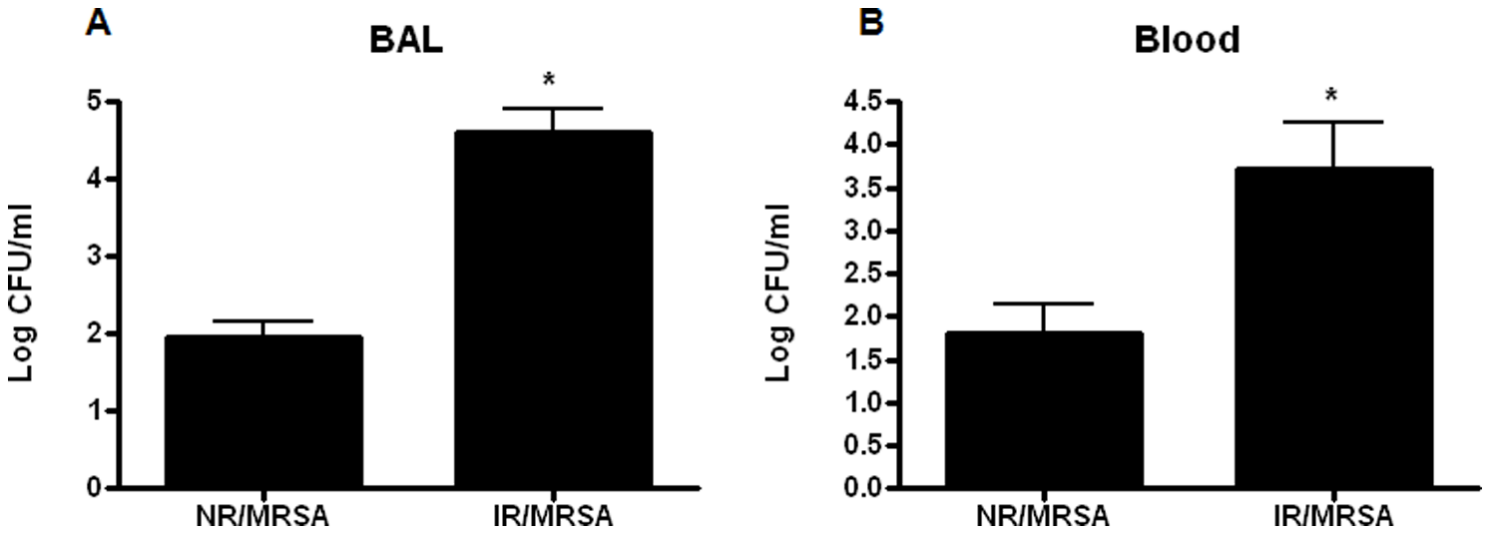
Radiation combined injury increases local and systemic infection following MRSA pneumonia. Bacterial colony counts were measured in BAL fluid (A) and blood (B) from mice subjected to MRSA pneumonia alone or radiation combined injury (n = 9−10/group). Animals subjected to IR/MRSA had higher levels of MRSA recovered from BAL fluid (p<0.05) and blood (p<0.001) 24 hours after induction of pneumonia.

IR/MRSA mice had a significant increase in MRSA cultured from BAL fluid compared to NR/MRSA mice (Fig. 2A). A similar increase in MRSA was detected in blood from IR/MRSA mice compared to NR/MRSA mice (Fig. 2B). Of note, mice given sham pneumonia did not have their BAL fluid or blood cultured based upon previous experience that sham operation does not cause detectable bacteria in either location (unpublished observations).

### IR and MRSA each Cause Leukopenia in Isolation, but there is Minimal Synergistic Effect of Radiation Combined Injury

To determine the impact of IR alone, MRSA alone and radiation combined injury on white blood cell count and differential, blood was sampled just prior to MRSA and in the same animals 24 hours later (Fig. S1A–C). IR alone led to a significant decrease in total white blood count (WBC), absolute neutrophil count (ANC), and absolute leukocyte count (ALC) compared to NR mice. MRSA pneumonia alone also caused a significant decrease in WBC count in NR mice which was exclusively due to a decrease in ALC, as ANC was not altered by MRSA pneumonia. Mice subjected to IR/MRSA did not have a significant change in WBC or ALC from mice subjected to IR alone (although ANC was higher in IR/MRSA mice), suggesting there was not a large synergistic effect of radiation combined injury.

### IR but not MRSA Causes a Decrease in Splenic Immune Populations, which is Exacerbated in Radiation Combined Injury

**Figure 3:**
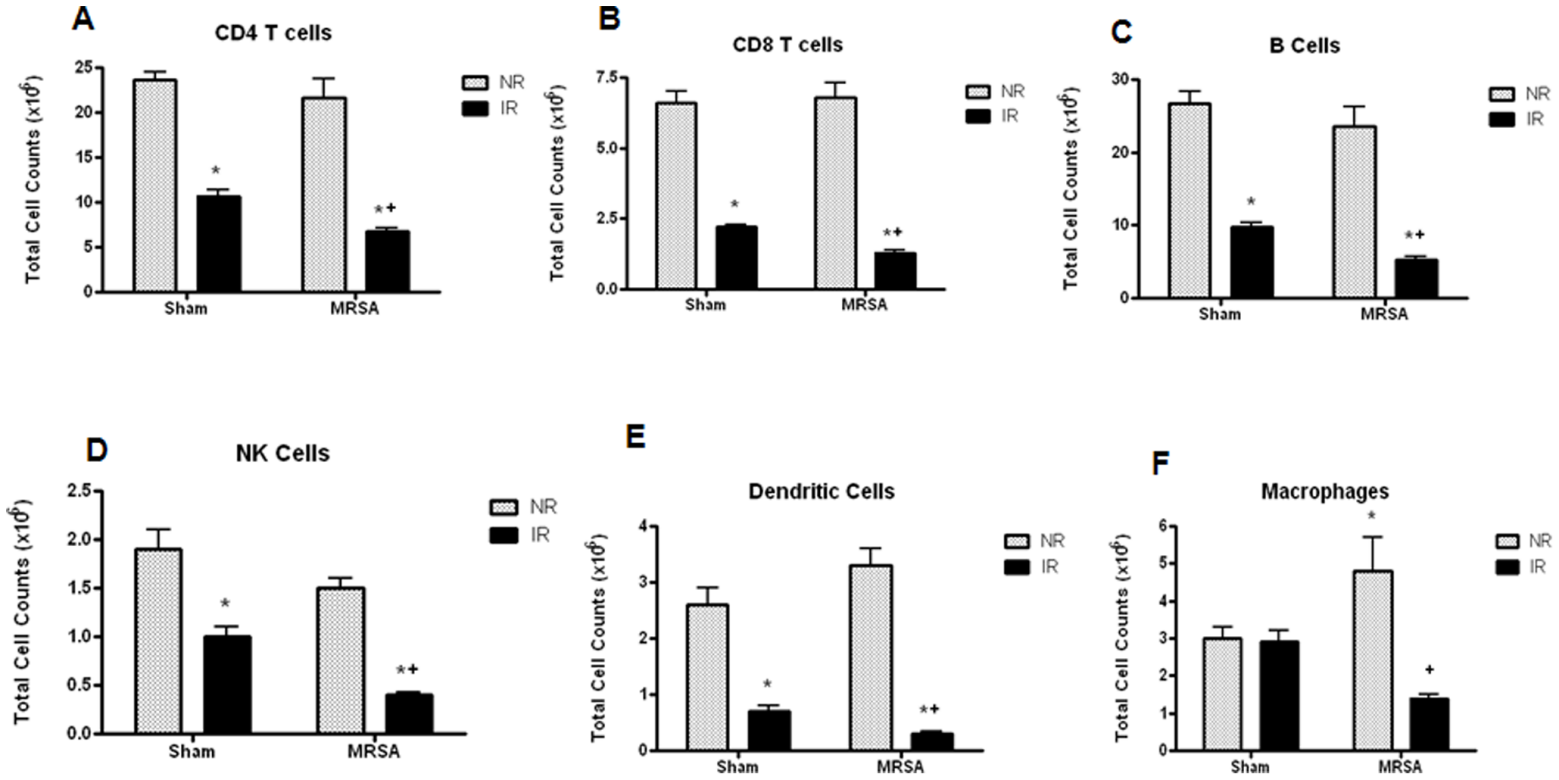
Radiation combined injury worsens the decrease in splenic immune populations seen following IR. Isolated splenocytes were stained for CD4 T cells (A), CD8 T cells (B), B cells (C), NK cells (D), dendritic cells (E), and macrophages (F) to determine differential splenic cell counts (n = 9−12 IR/sham, NR/MRSA, IR/sham, n = 5 NR/sham). Mice subjected to IR alone had lower cell counts than NR/sham mice for all populations measured except macrophages (p<0.01 for all). Cell counts were further diminished in animals subjected to radiation combined injury (p<0.001 for CD4, p<0.0001 for CD8, B cells, NK cells, p<0.01 for dendritic cells comparing IR/MRSA to IR/sham).

In order to better characterize the effect of IR and MRSA pneumonia on different subsets of immune cells, splenocytes were isolated and stained for CD4 T cells, CD8 T cells, B cells, NK cells, dendritic cells, and macrophages. IR alone caused a significant decrease in each of these cell types except for splenic macrophages (Fig. 3). In contrast, MRSA alone had no effect on any cell population except for a modest increase in macrophages compared to sham animals. Radiation combined injury further exacerbated the defects seen in IR/sham mice with lower counts for all cell types examined except macrophages in IR/MRSA mice compared to IR/sham mice.

### IR but not MRSA Causes an Increase in Lymphocyte Apoptosis which is Unaffected by Radiation Combined Injury

In order to determine whether apoptosis was responsible for cell loss, splenic T and B cells were stained with activated caspase-3 or TUNEL (Fig. S2). IR alone induced a significant increase in apoptosis by both methods in both T and B cells, while MRSA alone had no effect. There was no synergistic impact of radiation combined injury as T and B cell apoptosis was similar between IR/sham mice and IR/MRSA mice.

### Effect of IR, MRSA and Radiation Combined Injury on Local and Systemic Cytokines

**Figure 4:**
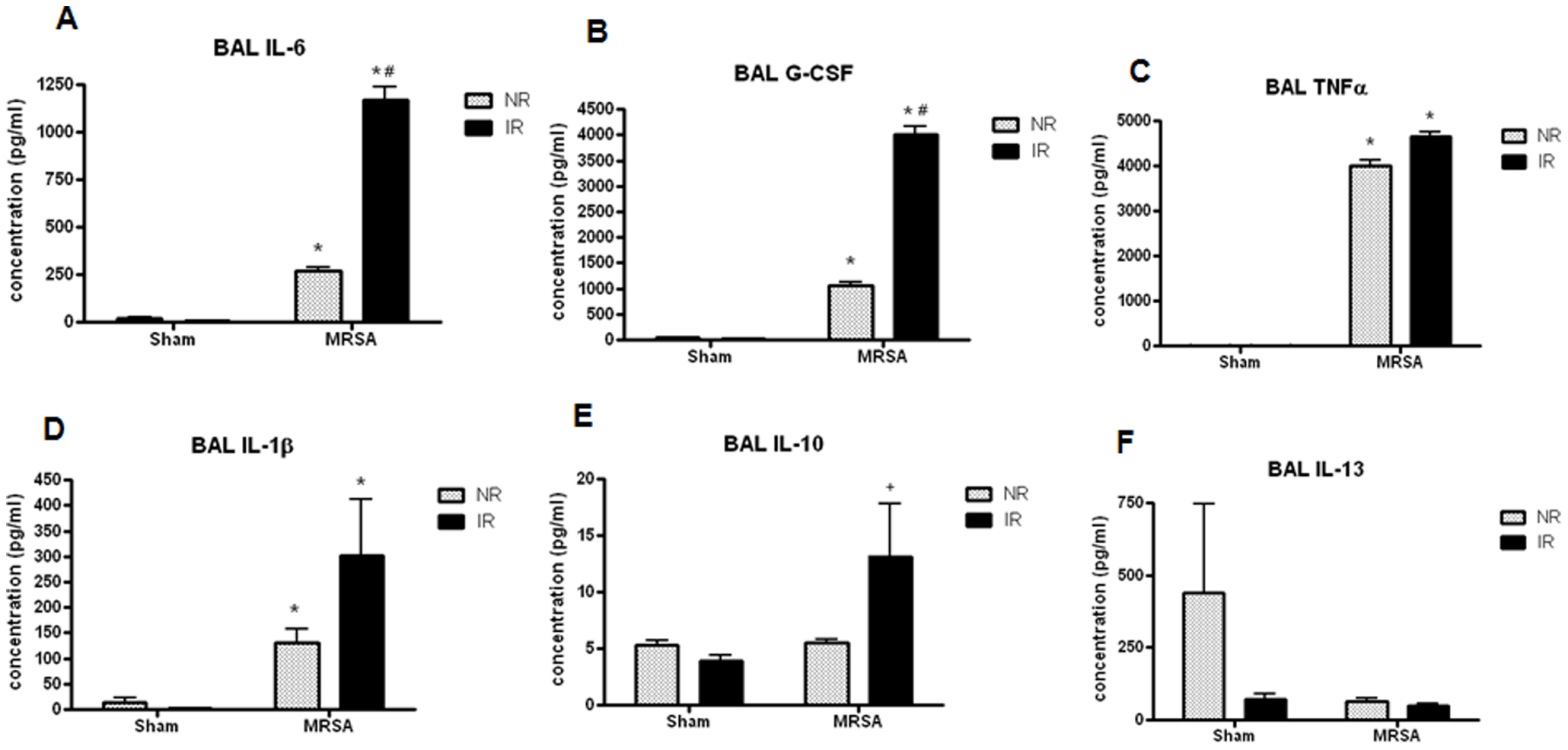
Radiation combined injury causes increased BAL IL-6 and G-CSF. MRSA pneumonia caused an increase in BAL IL-6, G-CSF, TNFα, IL-1β and IL-10 compared to NR/sham mice (p<0.001 for IL-6 and IL-1β, p<0.0001 for G-CSF, TNFα, and IL-10). A further increase in IL-6 and G-CSF (p<0.001) was seen in IR/MRSA mice compared to NR/MRSA animals (n = 12−17/group for al comparisons).

IR alone caused no statistically significant impact on pro- or anti-inflammatory cytokines in BAL fluid (Fig. 4). MRSA pneumonia induced an increase in IL-6, G-CSF, TNFα, IL-1β, and IL-10. Radiation combined injury led to a further augmentation in IL-6 and G-CSF, as levels of these cytokines were higher in IR/MRSA animals compared to NR/MRSA animals.

**Figure 5:**
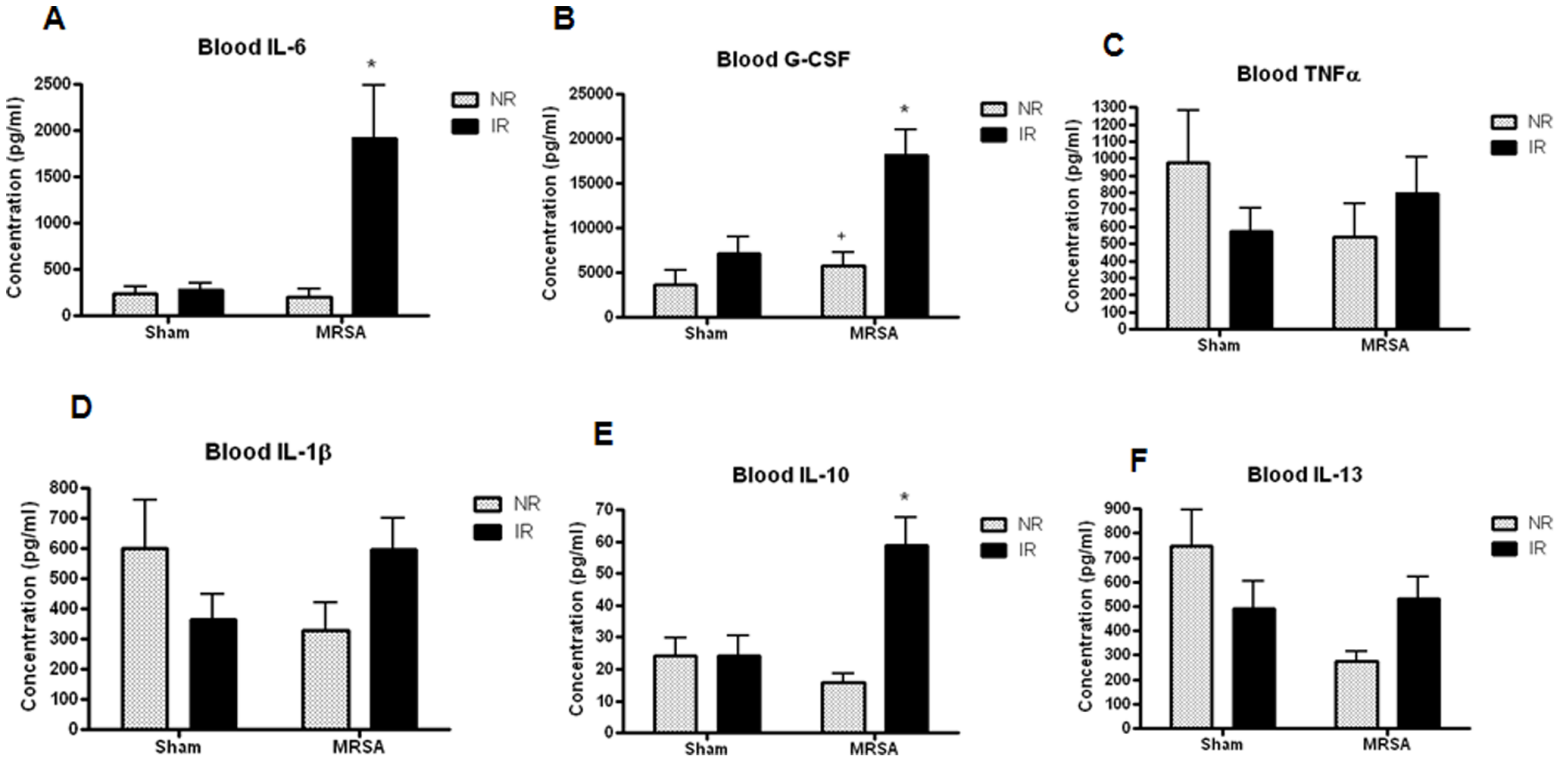
Radiation combined injury causes increased systemic levels of IL-6, IL-10 and G-CSF. While neither IR nor MRSA caused alterations in systemic cytokines (except for an increase in G-CSF following MRSA), IL-6 (p<0.005), IL-10 (p<0.01) and G-CSF (p<0.05) were significantly increased in IR/MRSA mice compared to NR/sham, IR/sham, NR/MRSA mice (n = 10−14/group).

IR alone also had no statistically significant impact on pro- or anti-inflammatory cytokines in the serum (Fig. 5) while MRSA pneumonia alone led to only a small increase in G-CSF. In contrast, radiation combined injury let to a significant increase in IL-6 and IL-10 levels as well as a further augmentation of G-CSF levels.

### MRSA but not IR Causes Pulmonary Injury which is Unaffected by Radiation Combined Injury

IR alone caused no evidence of lung injury (Fig. S3A). Non-lethal intratracheal MRSA injection caused mild pneumonia. Pulmonary histology was not worsened by radiation combined injury as pneumonia severity was similar between NR/MRSA and IR/MRSA mice. Similar findings were found with pulmonary neutrophil infiltration as MRSA pneumonia yielded an increase in MPO activity which was not worsened in radiation combined injury (Fig. S3B). No difference in lung wet to dry ratio was noted in any animals, independent of whether they received IR, MRSA pneumonia or radiation combined injury (Fig. S3C).

### Radiation Combined Injury Causes a Marked Increase in Gut Epithelial Apoptosis Compared to IR or MRSA Pneumonia in Isolation

**Figure 6:**
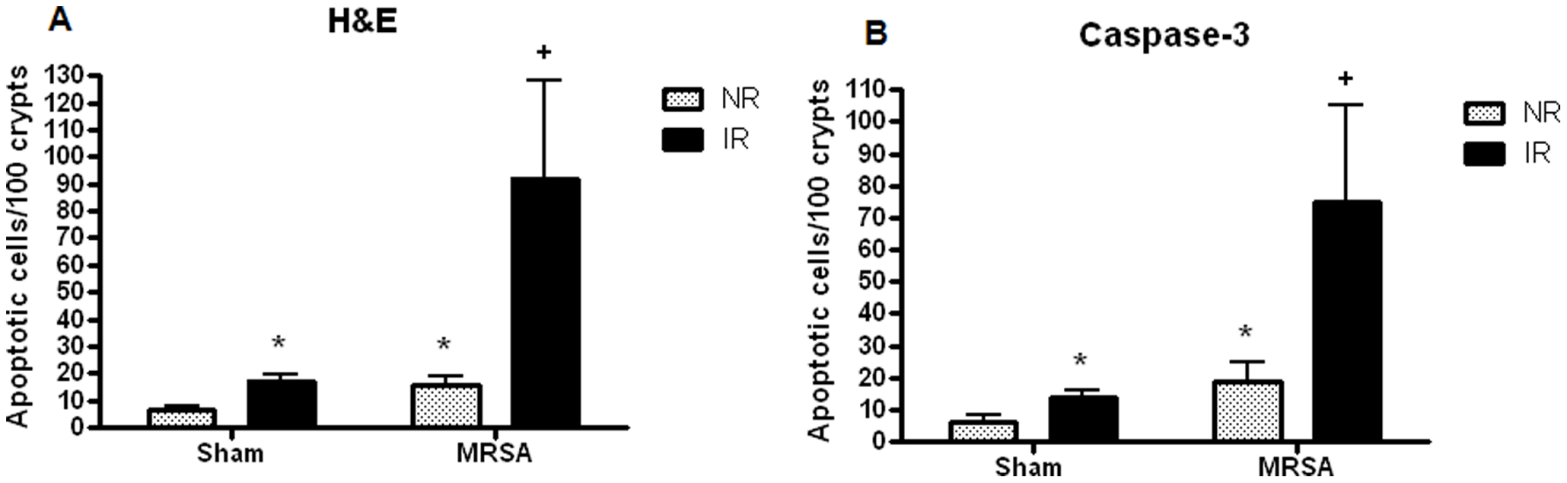
Radiation combined injury causes a marked increase in gut epithelial apoptosis. Gut apoptosis was modestly increased in both IR/sham and NR/MRSA mice compared to NR/sham mice both H&E (A) and active caspase-3 (B) staining (p<0.01 for both). Radiation combined injury induced a significant further increase in gut apoptosis as the number of apoptotic cells was 3–5 times higher in IR/MRSA mice compared to either injury in isolation (p<0.01 compared to all groups for H&E, p<0.05 compared to all groups for active caspase-3, n = 9–10/group).

Both IR alone and MRSA pneumonia alone caused a statistically significant increase in gut epithelial apoptosis, whether assayed by either H&E or active caspase-3 staining (Fig. 6). Radiation combined injury induced a further 3–5 fold upregulation in gut apoptosis, as IR/MRSA mice had a synergistic increase in apoptosis compared to either IR/sham or NR/MRSA mice.

In contrast to gut apoptosis, no difference in villus length or intestinal proliferation was noted in any animals, independent of whether they received IR, MRSA pneumonia or radiation combined injury (Fig. S4).

### Increased Gut Epithelial Apoptosis in Radiation Combined Injury Occurs Through the Mitochondrial Pathway

**Figure 7:**
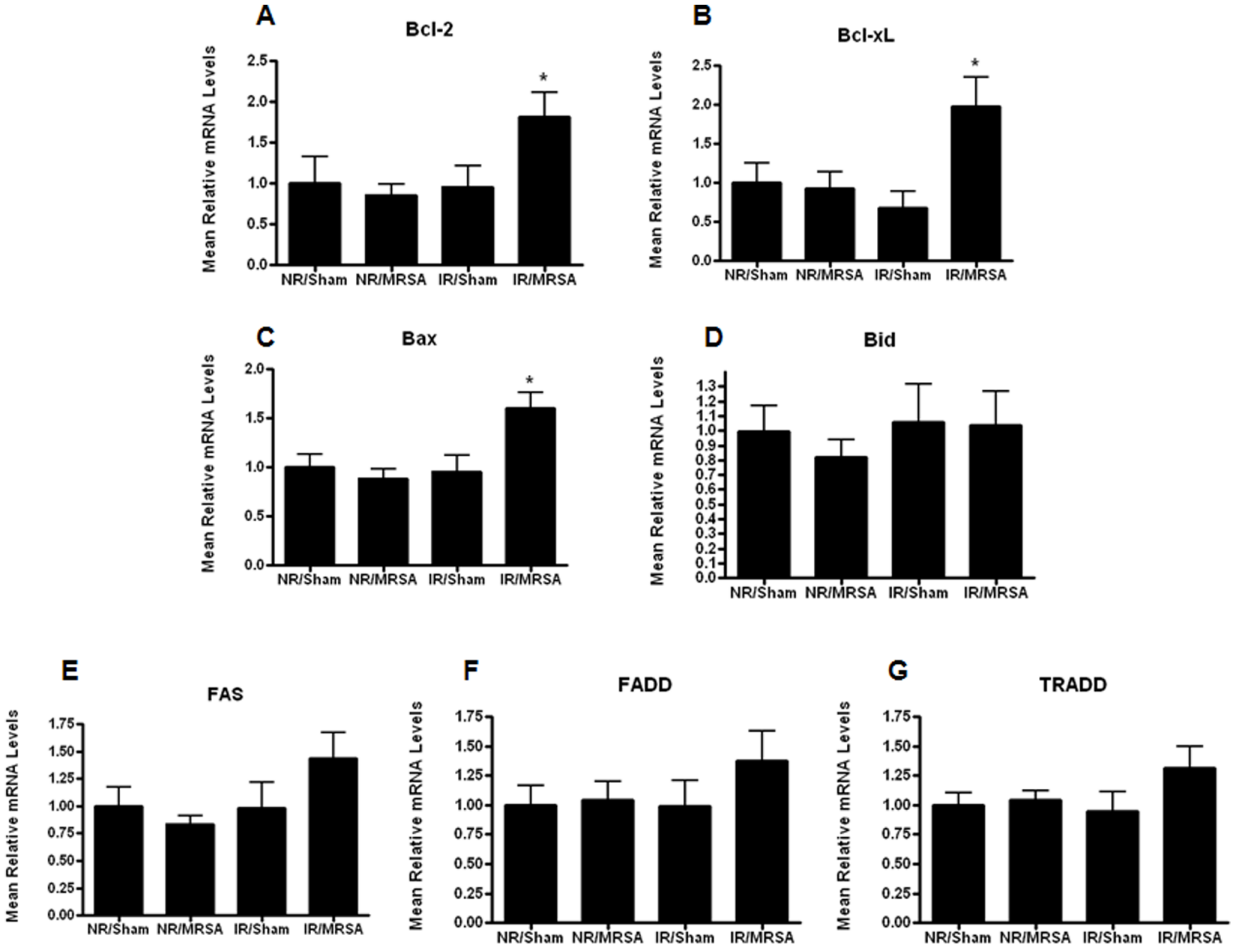
Increased gut apoptosis with radiation combined injury is associated with changes in the mitochondrial pathway. Real time PCR was performed on mediators in both the mitochondrial pathway and receptor-mediated pathway of apoptosis. Radiation combined injury caused an increase in mRNA levels of Bcl-2 (A), Bcl-x_L_ (B), and Bax (C, p<0.05 IR/MRSA compared to all other groups). In contrast, no alterations were noted in Bid (D), Fas (E), FADD (F) or TRADD levels (G, n = 7–8/group for all mediators).

To determine potential pathways through which radiation combined injury augmented gut apoptosis, expression of mRNA of several apoptosis mediators from both the mitochondrial (Bcl-2, Bcl-X_L_, Bax, Bid) and receptor-mediated (FAS, FADD, TRADD) pathways were measured using real-time PCR. Despite the modest increase in gut apoptosis caused by both IR alone and MRSA pneumonia, neither of these insults in isolation induced a statistically significant change in any apoptotic mediator (Fig. 7). However, radiation combined injury impacted the mitochondrial pathway as there were increases in both pro- (Bax) and anti-apoptotic mediators (Bcl-2 and Bcl-X_L_) in animals subjected to IR/MRSA. In contrast, radiation combined injury did not affect any mediators in the receptor-mediated pathway.

### Preventing Intestinal Epithelial Apoptosis Improves Survival Following Radiation Combined Injury

**Figure 8:**
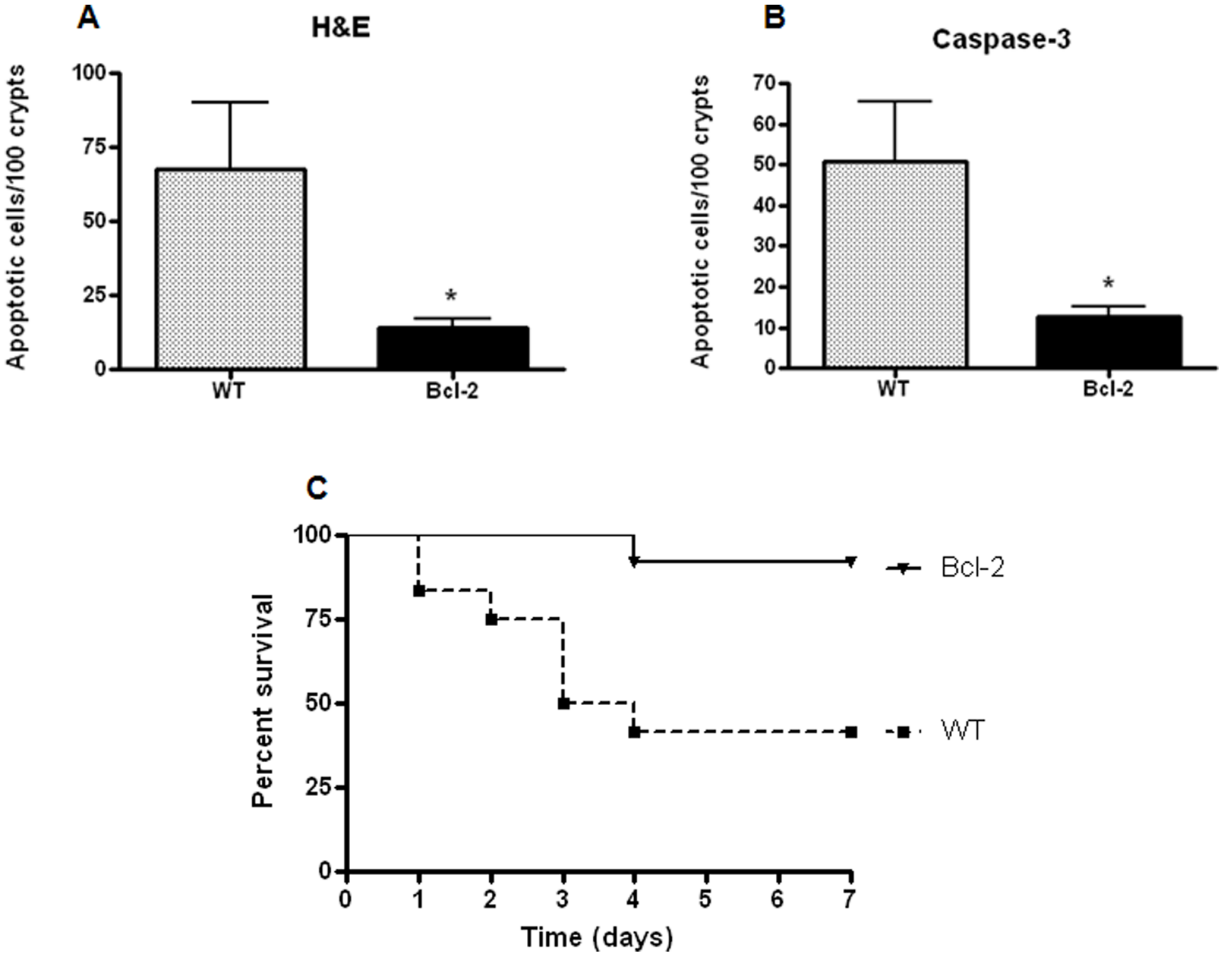
Gut Bcl-2 overexpression prevents intestinal epithelial apoptosis and improves survival following radiation combined injury. Gut apoptosis was lower in *Fabpl*-Bcl-2 mice than WT mice following IR/MRSA by both H&E (A) and active caspase-3 (B) staining (p<0.05 for H&E, p<0.005 for active caspase-3, n = 12–15). Preventing gut apoptosis led to improved outcome, as survival was 92% in *Fabpl*-Bcl-2 mice subjected to radiation combined injury compared to 42% in WT mice given the same insult (p<0.01, n = 12–13/group). Note that day 0 in this survival curve represents when MRSA pneumonia was induced, and IR was given four days earlier.

Since increased gut apoptosis was associated with alterations in the mitochondrial pathway in radiation combined injury, transgenic mice that overexpress Bcl-2 in their gut epithelium (*Fabpl*-Bcl-2) and wild type (WT) mice were subjected to IR/MRSA. *Fabpl*-Bcl-2 had a marked decrease in gut apoptosis following radiation combined injury compared to WT mice subjected to the same insult (Fig. 8A, B). Notably, prevention of gut apoptosis led to a marked benefit in mortality, with survival increasing from 42% in WT mice to 92% in *Fabpl*-Bcl-2 mice (p<0.01, Fig. 8C).

Since radiation combined injury induces an upregulation in BAL and systemic bacterial burden and cytokines compared to IR or MRSA alone (Figs. 2, 4 and 5), *Fabpl*-Bcl-2 and WT mice were subjected to IR/MRSA to determine whether the survival advantage conferred by preventing gut apoptosis secondarily impacted these extra-intestinal variables. No significant differences were detected between *Fabpl*-Bcl-2 and WT mice in local or systemic bacteremia (Fig. S5) or cytokines that were impacted by radiation combined injury (Fig. S6).

## Discussion

This study demonstrates that even though both low-dose IR and low-dose MRSA infection are non-lethal, radiation combined injury with IR/MRSA results in nearly 50% mortality. Compared to IR or MRSA alone, mice with radiation combined injury have increased gut apoptosis, increased bacteria recoverable from both BAL fluid and blood, a decrease in all splenic immune cells assayed (except for macrophages) and increased BAL and systemic IL-6 and G-CSF. Notably, survival was nearly 100% following IR/MRSA when gut apoptosis was prevented by intestine-specific overexpression of Bcl-2 in transgenic mice.

Both IR and sepsis cause significant organ damage in isolation, with the degree of injury related to degree of radiation exposure or severity of sepsis. Acute lethality following whole body IR is dependent on severity of exposure – mild (1–2 Gy) causes no mortality while doses above 8–10 Gy are lethal [32]. Even at very low doses, IR causes host damage, disproportionately affecting cells with a high turnover rate such as the hematopoietic system and the gastrointestinal system [33]–[35]. Similarly, sepsis causes immunosuppression, increased gut and immune apoptosis and organ dysfunction [36]–[38]. However, the functional significance of abnormalities associated with either injury in isolation at the doses given in this study appears to be relatively limited, since neither IR nor MRSA alone were sufficient to cause mortality.

In contrast, mortality was markedly increased in IR/MRSA mice, consistent with radiation combined injury models by other groups using burn, trauma, gram negative bacteria or enteric contents as the second injury [5]–[9]. To determine possible mechanisms of mortality, we performed a thorough analysis comparing NR/sham, IR/sham, NR/MRSA and IR/MRSA mice to be able to delineate which abnormalities observed were due to IR alone, MRSA alone, or a synergistic effect seen with radiation combined injury.

The intestine was a logical place to focus on in light of the fact that the gut has been shown to be a central player in both a) radiation sickness where proliferating crypt cells are exquisitely radiosensitive [33], [39] and b) sepsis, where the gut has often been hypothesized to be the “motor” of the systemic inflammatory response with significant alterations in gut integrity seen in various infections including MRSA [21], [40]–[42]. Our results demonstrated a massive upregulation in gut apoptosis following radiation combined injury, significantly higher than could have been predicted from the increase in apoptosis seen with either IR or MRSA alone. To determine the functional significance of this finding, it was first necessary to determine pathways through with IR/MRSA induced gut apoptosis. This was unclear in light of the fact that IR has been shown to induce gut apoptosis through the mitochondrial pathway [39], [43], [44] whereas MRSA induces gut apoptosis through both the mitochondrial and receptor-mediated pathways [21]. We found that radiation combined injury appears to induce gut apoptosis dominantly through the mitochondrial pathway, since only Bax, Bcl-2 and Bcl-X_L_ were altered in IR/MRSA. Based upon this information, *Fabpl*-Bcl-2 and WT mice were subjected to IR/MRSA with the former having a marked survival advantage. These results suggest that gut apoptosis may be an attractive therapeutic target in radiation combined injury. We are unaware of any agents that specifically target Bcl-2 that could be given as a therapeutic agent to prevent gut apoptosis. However, systemic epidermal growth factor has been shown to prevent sepsis-induced mortality and gut apoptosis in an intestine-dependent fashion following either peritonitis or *Pseudomonas aeruginosa* pneumonia [30], [45], [46] and thus represents a potential therapeutic agent in radiation combined injury. Of note, intestinal damage in IR/MRSA appears to be restricted, at least in part, to apoptosis since neither gut proliferation nor villus length was affected by radiation combined injury. Notably, *Fabpl*-Bcl-2 mice subjected to radiation combined injury did not have significant differences in either local or systemic bacterial burden or cytokine levels compared to WT mice given the same insult. This stands in distinction to the observation that each of these physiological variables was worsened (increased bacterial burden, increased pro-inflammatory cytokines in blood and BAL) in mice subjected to radiation combined injury compared to IR or MRSA alone. This suggests that neither bacterial burden nor cytokines were responsible for the survival advantage conferred by prevention of gut apoptosis, and the mechanism through which preventing gut apoptosis improves survival advantage requires further study. However, this does not preclude a role of either bacterial translocation or inflammation in mediating mortality in radiation combined injury compared to IR or MRSA alone via an additional mechanism independent of gut apoptosis, as it is certainly plausible that more than one mechanism is responsible for the decreased survival seen following radiation combined injury.

As the hematopoietic system is also sensitive to both IR and sepsis, we examined the spleen and blood in radiation combined injury. While IR in isolation decreased all cell populations in the spleen except for macrophages, a further decrease was noted in CD4 T cells, CD8 T cells, B cells, NK cells, and dendritic cells following IR/MRSA. This likely caused immunosuppression and may be, in part, responsible for the increased bacterial burden seen in animals subjected to IR/MRSA compared to either insult in isolation. Interestingly, the decrease in splenic lymphocytes was not associated with an increase in lymphocyte apoptosis. This suggests that alterations in migration, production, or other types of cell death may be responsible, although we cannot rule out that our single timepoint snapshot failed to capture a true alteration in apoptosis. The finding that radiation combined injury led to increased splenic subpopulation loss compared to IR contrasts with the results of Tajima et al. which showed slightly higher numbers in mice that underwent IR with combined burn injury. Possible explanations for our disparate findings include different secondary injury (MRSA vs. burn), inbred vs. outbred mice (FVB/N vs. CD-1) and different doses of radiation (2.5 Gy vs. seven doses ranging from 0–8 Gy but no dose of 2.5 Gy tested). Additionally, our findings indicated that IR caused a profound systemic leukopenia, with decreases in WBC, ANC and ALC. However, unlike in the spleen, decreases in blood WBC was not exacerbated in radiation combined injury, suggesting that immune cell populations respond differently depending upon which compartment they are in.

Since lung is the primary site of injury with MRSA pneumonia and can be a site of damage following IR [47], it was logical to examine the lungs in this study. Globally, the lungs were modestly impacted by MRSA, with mild pneumonia and increased MPO activity, but without differences in wet to dry ratio. This is consistent with the nonlethal nature of intratracheal MRSA injection. The fact that pulmonary injury was not exacerbated in IR/MRSA mice suggest that the lungs are not a major cause of mortality in the radiation combined injury model used.

This study has several limitations. First, all mechanistic parameters were examined at a single timepoint –24 hours – after either MRSA or sham pneumonia. This represents a “snapshot” of the parameters studied and prevents further insights that might be obtained by sacrificing animals at multiple times after the second insult. We also did not examine any variables shortly after IR in light of the large existing literature on the effects of IR in isolation. Rather nearly all variables examined in IR/sham and IR/MRSA mice were obtained 5 days after IR (24 hours after MRSA or sham pneumonia) so early effects of IR would not be detected by our study design. Additionally, animals were followed for only seven days after MRSA or sham pneumonia (a total of 11 days after IR). Recent literature suggests that death occurs up to one month after alternative models of murine sepsis such as cecal ligation and puncture [48], and stopping our survival curve at seven days precluded determining if late deaths occurred in any of the groups examined. Another limitation is the timepoint at which MRSA itself was given. Since both the gut and the hematopoietic system have rapid turnover, it is likely that giving MRSA at a different interval after IR would have resulted in different findings, which is potentially significant since it is unclear when the peak incidence of secondary infection would be in radiation combined injury. Additionally, the alterations in the immune system, cytokines and bacterial burden in radiation combined injury compared to IR or MRSA alone are associative. While each of these may play a role in the increased mortality seen following radiation combined injury, further studies need to be performed to determine if they have functional significance. While IR was always given between 9 am and 4 pm, there is a strong circadian response to radiation exposure. In each experiment, IR was performed at the same time in different groups (i.e. IR vs. IR/MRSA in WT mice or *Fabpl*-Bcl-2 vs., WT mice for IR/MRSA) which should have minimized intergroup variability; however, we cannot rule out that inter-experiment variability occurred due to altering what time IR was given when multiple replicates were combined. In addition, the use of a surgical procedure for intratracheal injection of MRSA or saline represents a potential confounder as incisional wounds following IR could represent another model of radiation combined injury, although no incisional wounds were identified throughout the course of the study.

Despite these limitations, these results demonstrate that IR/MRSA results in far greater mortality than could have been expected in isolation and this is associated with alterations predominantly in cell types with high turnover. Preventing gut apoptosis minimizes mortality in a murine model of radiation combined injury and may represent a therapeutic approach in case of a terrorist attack or nuclear accident. Since it is not feasible to perform human trials of radiation combined injury, further mechanistic and functional animal studies should be undertaken to determine whether systemic agents that prevent gut apoptosis may be appropriate downrange for licensure for biodefense countermeasures.

## Supporting Information

Figure S1 **Radiation combined injury has minimal affect on leukopenia caused by both IR and MRSA.** Animals (n = 7−9/group) had blood drawn just prior to MRSA pneumonia or 24 hours later for total white blood cell count (A), absolute neutrophil count (B) or absolute lymphocyte count (C). Both IR alone (p<0.001) and MRSA (p<0.05) decreased white blood cell count. IR also decreased ANC (p<0.01) but this was not impacted by MRSA. ALC was also lower following both IR (p<0.001) and MRSA (p = 0.001). Compared to mice given IR alone, radiation combined injury did not impact WBC or ALC and actually increased ANC (p<0.01).
(TIF)

Figure S2 **Radiation combined injury has minimal affect on splenic T and B lymphocyte apoptosis caused by IR.** Isolated splenic T cells (A,B) and B cells (C,D) were stained with either activated caspase -3 (A, C) or TUNEL (B, D) to measure lymphocyte apoptosis (n = 9−12 IR/sham, NR/MRSA, IR/sham, n = 5 NR/sham). Mice subjected to IR alone had increased T and B cell apoptosis (p<0.05 compared to NR/sham for all except P<0.01 for caspase-3 in T cells) but this was not augmented in mice subjected to IR/MRSA.
(TIF)

Figure S3 **Radiation combined injury has minimal affect on pulmonary injury caused by MRSA.** Pneumonia severity (A) was mildly increased in mice subjected to MRSA (p<0.05 compared to NR/sham) but was not augmented in animals given IR/MRSA(n = 8−9/group) using a severity score that ranged from 0 (no histopathologic signs of pneumonia) to 4 (severe pneumonia). Similar results were found for MPO activity (B) where neutrophil infiltration was increased in mice subjected to MRSA (p<0.01 compared to NR/sham) but was not augmented in animals given IR/MRSA (n = 5−6/group). Wet-to-dry lung ratio (C) was similar in all groups (n = 9−10/group).
(TIF)

Figure S4 **Radiation combined injury has minimal affect on villus length or intestinal permeability.** Villus length (A) was measured from the crypt neck to the villus tip and was similar in all groups regardless of whether they were subjected to IR, MRSA or radiation combined injury (n = 9−15/group). Crypt proliferation (B) was also similar in all groups (n = 9−15/group).
(TIF)

Figure S5 **Gut Bcl-2 overexpression does not affect local and systemic infection following radiation combined injury.** Bacterial colony counts of MRSA were similar in both BAL fluid (A) and blood (B) from *Fabpl*-Bcl-2 and WT mice following IR/MRSA (n = 5−15/group).
(TIF)

Figure S6 **Gut Bcl-2 overexpression does not affect local or systemic cytokines following radiation combined injury.** Cytokines that were increased by radiation combined injury (figure 6 and 7) were compared in *Fabpl*-Bcl-2 and WT mice following IR/MRSA and found to have similar concentrations (n = 13−17/group for BAL cytokines and n = 7−19/group for systemic cytokines).
(TIF)
