# Supplementary figures and images for: Inhibition of Intestinal Epithelial Apoptosis Improves Survival in a Murine Model of Radiation Combined Injury
Source: PLoS One. 2013 Oct 28;8(10):e77203. doi: 10.1371/journal.pone.0077203 (PMC3810465; doi:10.1371/journal.pone.0077203)

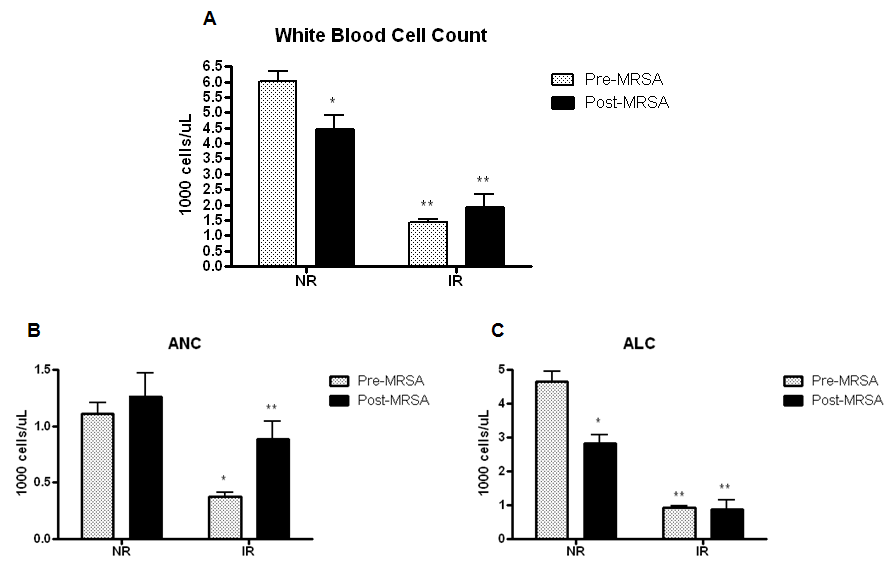

Supplement: Figure S1 — Radiation combined injury has minimal affect on leukopenia caused by both IR and MRSA. Animals (n = 7−9/group) had blood drawn just prior to MRSA pneumonia or 24 hours later for total white blood cell count (A), absolute neutrophil count (B) or absolute lymphocyte count (C). Both IR alone (p<0.001) and MRSA (p<0.05) decreased white blood cell count. IR also decreased ANC (p<0.01) but this was not impacted by MRSA. ALC was also lower following both IR (p<0.001) and MRSA (p = 0.001). Compared to mice given IR alone, radiation combined injury did not impact WBC or ALC and actually increased ANC (p<0.01). (TIF) [file pone.0077203.s001.tif]

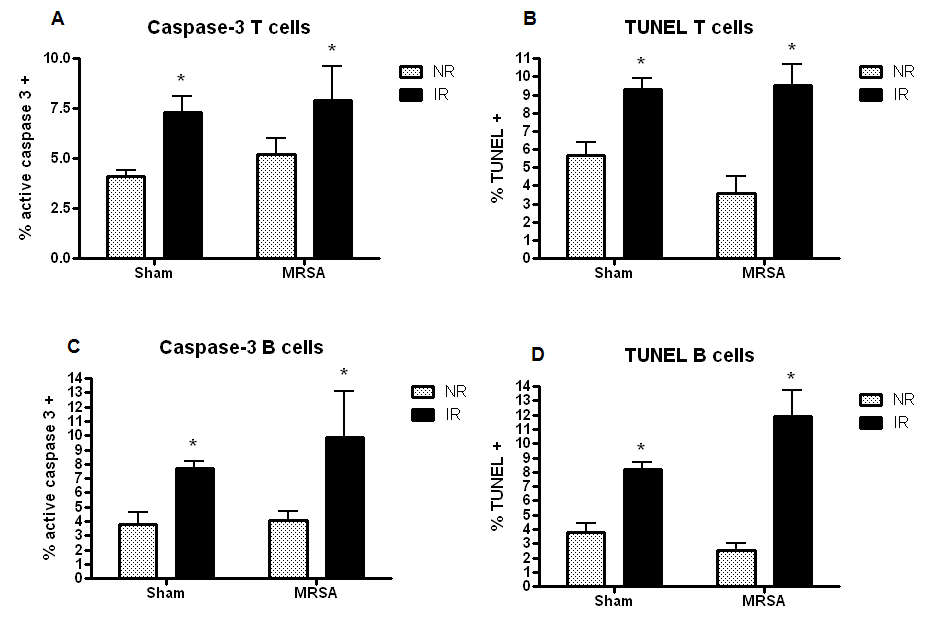

Supplement: Figure S2 — Radiation combined injury has minimal affect on splenic T and B lymphocyte apoptosis caused by IR. Isolated splenic T cells (A,B) and B cells (C,D) were stained with either activated caspase -3 (A, C) or TUNEL (B, D) to measure lymphocyte apoptosis (n = 9−12 IR/sham, NR/MRSA, IR/sham, n = 5 NR/sham). Mice subjected to IR alone had increased T and B cell apoptosis (p<0.05 compared to NR/sham for all except P<0.01 for caspase-3 in T cells) but this was not augmented in mice subjected to IR/MRSA. (TIF) [file pone.0077203.s002.tif]

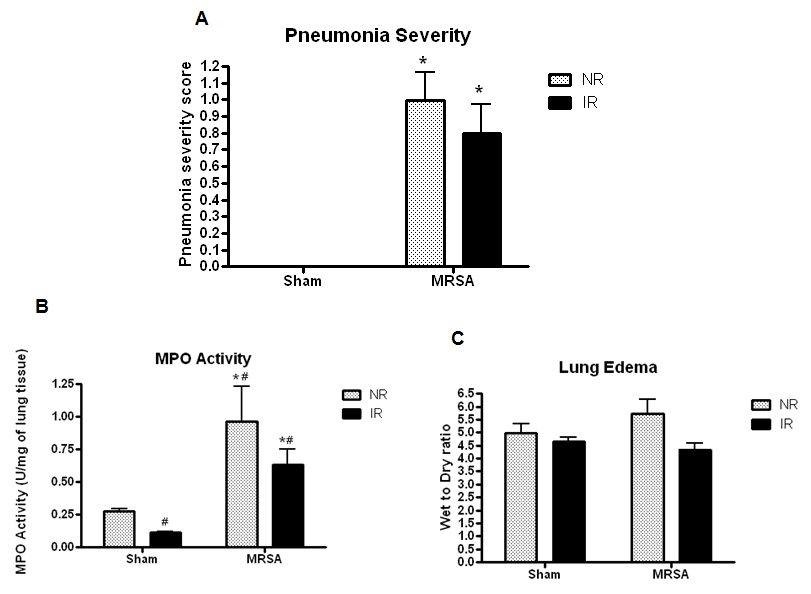

Supplement: Figure S3 — Radiation combined injury has minimal affect on pulmonary injury caused by MRSA. Pneumonia severity (A) was mildly increased in mice subjected to MRSA (p<0.05 compared to NR/sham) but was not augmented in animals given IR/MRSA(n = 8−9/group) using a severity score that ranged from 0 (no histopathologic signs of pneumonia) to 4 (severe pneumonia). Similar results were found for MPO activity (B) where neutrophil infiltration was increased in mice subjected to MRSA (p<0.01 compared to NR/sham) but was not augmented in animals given IR/MRSA (n = 5−6/group). Wet-to-dry lung ratio (C) was similar in all groups (n = 9−10/group). (TIF) [file pone.0077203.s003.tif]

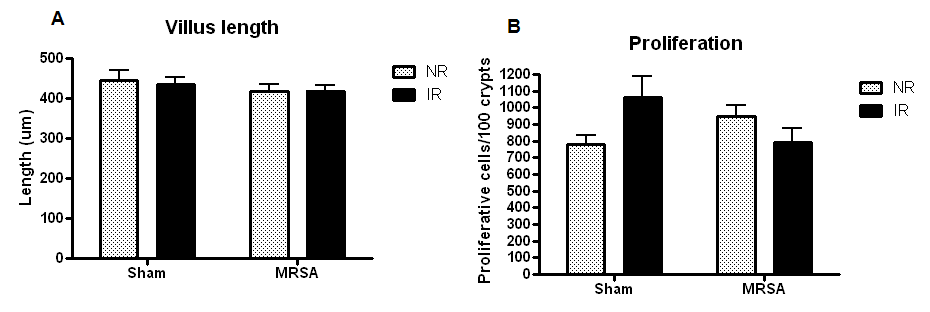

Supplement: Figure S4 — Radiation combined injury has minimal affect on villus length or intestinal permeability. Villus length (A) was measured from the crypt neck to the villus tip and was similar in all groups regardless of whether they were subjected to IR, MRSA or radiation combined injury (n = 9−15/group). Crypt proliferation (B) was also similar in all groups (n = 9−15/group). (TIF) [file pone.0077203.s004.tif]

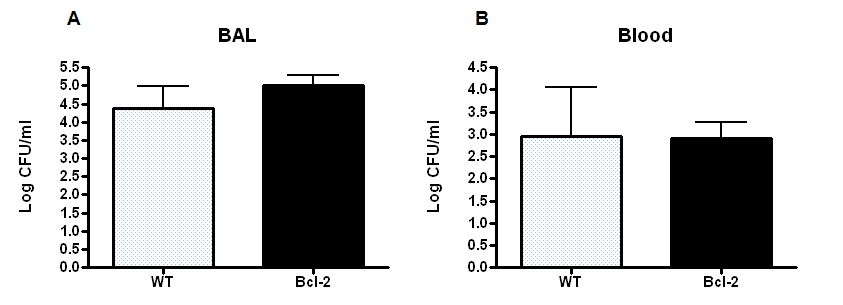

Supplement: Figure S5 — Gut Bcl-2 overexpression does not affect local and systemic infection following radiation combined injury. Bacterial colony counts of MRSA were similar in both BAL fluid (A) and blood (B) from Fabpl-Bcl-2 and WT mice following IR/MRSA (n = 5−15/group). (TIF) [file pone.0077203.s005.tif]

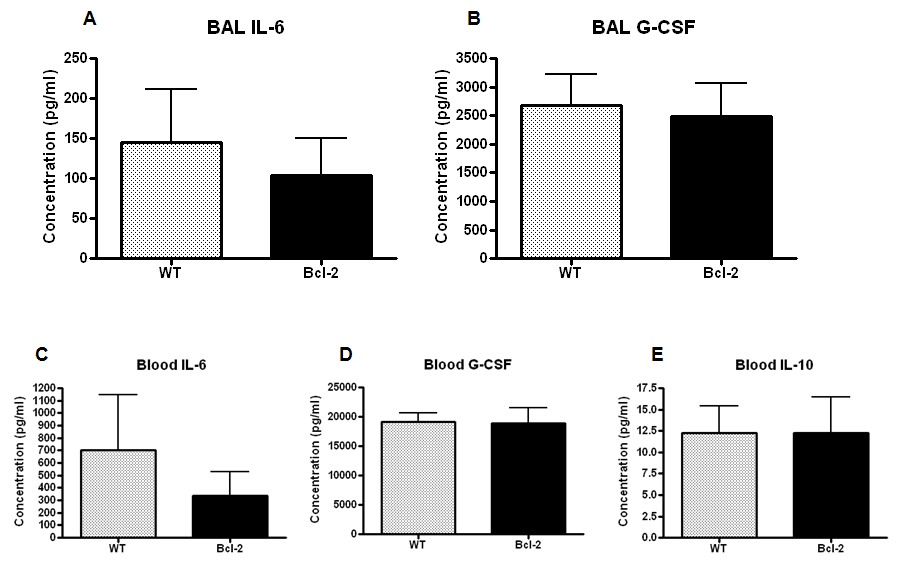

Supplement: Figure S6 — Gut Bcl-2 overexpression does not affect local or systemic cytokines following radiation combined injury. Cytokines that were increased by radiation combined injury (figure 6 and 7) were compared in Fabpl-Bcl-2 and WT mice following IR/MRSA and found to have similar concentrations (n = 13−17/group for BAL cytokines and n = 7−19/group for systemic cytokines). (TIF) [file pone.0077203.s006.tif]
